# Neighborhood and physical activities of Portuguese adolescents

**DOI:** 10.1186/1479-5868-7-33

**Published:** 2010-05-05

**Authors:** Nuno Loureiro, Margarida G Matos, Maria M Santos, Jorge Mota, José A Diniz

**Affiliations:** 1 Escola Superior de Educação de Beja (ESEB), Beja, Portugal; 2 Faculdade de Motricidade Humana, Cruz Quebrada, Portugal; 3 Faculdade de Desporto da Universidade do Porto, Porto, Portugal

## Abstract

**Background:**

This study examines associations between perceptions of neighbourhood environment and physical activity and sports within Portuguese adolescents.

**Methods:**

The sample consisted of 4,877 individuals of both genders, with an average age of 14 years. The instrument used was the Health Behavior School-aged Children questionnaire.

**Results:**

Perceptions of the neighbourhood being unsafe for children to play and having no place to spend leisure time were associated with lower levels of exercise among adolescents. The perceptions of the neighbourhood being unsafe for children to play (OR = 1.3, p < .005) and the fact of not having a place to spend leisure time (OR = 1.3, p < .005) p < .005) were associated with lower levels of exercise among adolescents. The perception of these variables is associated to a lower probability of exercising. The neighbourhood characteristics are more important to the practice of outdoor sports than of indoor sports.

**Conclusion:**

The perceptions of the neighbourhood may influence adolescent's physical activity and sports, in different ways.

## Introduction

The last decades have been characterized by thorough and diverse investigations in the sphere of Physical Activity (PA) where four areas can be clearly identified [1]. The first area dated before the 1970s is characterized by physiological investigations on the impacts of PA in the physical condition of the individual; a second area dated between the 1970s and the 1990s sums up epidemiological investigations which led the PA to be seen as a fundamental and priority health factor; a third area, belonging to the same time line as the prior, focused investigations on the main forms of promoting PA, and on a fourth area, dated from the beginning the XXI century, investigations centered on politics and environmental factors to promote health. The ecological model starts becoming a vital part in the promotion of the activity and so showing the interactions of people in their physical, social and cultural environment [2]. This is distinct model because it explicitly includes the environment, political variations and their inferences in the behavior of the individual.

The level of the variations in the ecological model of PA includes domains like the intra personal (biological and psychological), inter personal or cultural, organizational, the physical environment (natural and built) and politics (laws, rules, regulations and codes) [3].

The physical environment, particularly the place where people live, presents a set of features and characteristics that have been studied and discussed as potential barriers or facilitators to the practice of physical activity or sports for adolescents [4].

The neighborhood is considered a key item in the examination of outdoor physical practices, offering the opportunity to have non expressive forms of PA such as walking and riding a bicycle [5]. The possibility or the difficulty of children playing in the street has developed a new classification of type of child: children who spend their free time in places outside their residence are called "outdoor children," and those who occupy their time watching TV and playing computer are the "interior children." The house is perceived by children and teenagers as a safe place against the outside dangers, providing safety and comfort, which can be assumed as an important place for the practice of PA. Thus a child who is limited in his/her PA practices at home may be inclined to have low levels of practice [6].

In an extensive review of the literature [7] the living environment has been analyzed according to three items: the conditions of the residential environment, infrastructure and recreational transport infrastructure.

The conditions of the residential environment include positive and negative factors. The characteristics of the prior classification are the safety of the neighborhood, the security of the playing areas, the crime rate, social disorder, strange dangers, physical disorder and the weather conditions [8]. A safe place may be seen as a place where one can walk alone after night fall, a place that allows children to play in the street, where one can ask for a favor or for help or where people greet each other and stop to chat [9].

The urban design and its influence on the practice of physical activities of teenagers are still ambiguous [4]. One of the aspects that has been recently studied is the neighborhood design and it has been discovered that design is positively associated to the practice of PA. That is to say, there are many interesting things to see when you are walking [10].

The location of the neighborhood seems to be a factor to be taken into account in the promotion of PA. Many studies [11,12] have tried to quantify the distances teenagers consider accessible to facilitate their autonomous movement from home to the playing area. Although there is no consensus, the distance of 1184 meters [11,12] and less than 1 km [13] or distances covered in 15 minutes [11] facilitate the engagement with physical activity. The students whose houses are closer to the school [14] or that are up to 800 meters from the school [15] have a greater possibility of going on foot.

The recreational infrastructure (playing area) for children and teenagers may be classified as private (in their house) or public (common areas or school areas) and private/public (commercial game areas) [8]. Besides their typology or classification, the existence of playing areas [16-20] and its proximity to the children's and teenager's home [16,17,21] is positively linked to the sports practice. For Evenson et al [22] the girls aware of sports facilities closer to home are twice as more active as their counter parts with the opposite perception and in the case of Caucasian girls it depends significantly more on the MET (measure unit for energy given up) [12].

The security of the parks [23] and its quality [24-26] seem to be very important factors for the practice of PA. On the other hand, the investigation done by Sallis, Taylor, Dowda, Freedson, e Pate [27] with 781 American adolescents reveals that there was no link found between the access to the parks, playing areas and gymnasiums with the sole aim of physical activity. This significance was not associated to the practice of PA in adolescents who live in flats, unsafe neighborhoods and whose families have very few resources [23]. There was also no link found between the proximity of the installations and the quantity of time spent in sedentary activities [28]. The place size seems not to condition the practice of PA [13].

The transport infrastructures are usually grouped in accordance with the existence of transport routes (e.g. routes for bicycles, fitness areas) and the dangers on the streets [8]. In some countries these infrastructures are of the responsibility of many organizations, like in the *Metropolitan Planning Organization *(MPO) in the USA, which define guidelines and projects that contribute to the mobility of its area habitants [29].

The bicycle routes are a very good example of infrastructures that may facilitate the mobility of the teenagers, but the studies do not present consensual and unanimous results [4].

The perception of danger in the passage routes where teenagers circulate is an important factor to be taken into account. These dangers may be perceived by the quantity of traffic or the many parking areas, which leads to the perception that the place is less pleasant to live in [9]. In the places where traffic is less problematic there is a perception that it is easier to walk or ride a bicycle [24]. This aspect is very evident in the studies by Timperio et al. [16] in which the perceptions of the parents on the amount of roads which their children have to cross to reach the playing areas and the lack of illumination and zebra crossing signs are negatively linked to the practice of PA of teenagers.

The main objective of the investigation is to understand the importance of residential neighborhood in the practice of different physical activities and sports of the Portuguese teenagers.

## Methodology

The present study used the data from the 2006 Portuguese sample of the Health Behavior in School-Aged Children (*HBSC*) [30]. This is the 2006 international investigation that took place in 44 countries and the main objectives consist in studying and monitoring the lifestyles of adolescents and their behaviors in the distinct social contexts.

### Sample

The sample is made up of 4877 individuals of 136 schools randomly selected from a national list stratified by region. The chosen unit is the class. It is a representative sample of students of these scholar levels in public schools of Portugal. So 50,4% (2460) of the participants are females and 49,6% (2417) belong to the male gender, presenting an average age of 14 years old (± *DP *1.89 for the age), varying between the minimum of 10 years and the maximum of 20 years old. Concerning the school grade, 31.7% (1546) are in the 6th grade, 35.7% (1740) the 8th grade and 32.6% (1591) the 10th grade.

### Instrument

The instrument used was the Health Behavior School-aged Children questionnaire. The participating countries in the HBSC study included all the compulsory items on the questionnaire which focus on distinct health aspects: at a demographic, behavior and psycho social level. All the questions followed the protocol format [31], focusing on demographic questions (age, gender, social and economical status), questions based on positive health, alcohol consumption, tobacco and drugs, physical activity, sexual behavior, injuries and violence, family, pair groups and leisure.

### Variables

The object of the study is to understand the importance of the residential neighborhood in the practice of different physical activities and sports of Portuguese teenagers. The questions are shown on table 1.

**Table 1:** Key Questions of the Questionnaire used in research and its codification

Questions	cod
1 - Are you boy or girl?	a) Boy; b) Girl

2 - Wich school grade do you attend?	a)6th, b) 8th; c) 10th

13 - Over the past seven days, on how many days were you physically active for a total of at least 60 minutes per day?	from 0 days to 7 days

73 - Outside of school, during your free time, how often do exercise or sports activities enough to keep the wheezing breath and sweating?	a) Every day b) 4 to 6 times/week. c) 2 to 3 times/week. d) 1 time/week, and) 1 time per month f) less than 1 time per month; g ) never

76 - What sports practice or practices during the last 6 months on a regular basis and outside of school physical education (at least 1 hour, twice a week)?	a) Soccer b) Basketball c) Gymnastics d) Volleyball e) Swimming, f) Cycling/Mountain Biking; g) Handball h) Athletics

97) Read the sentences below and notes about what you think regarding the place where you live: a) Do people get along well and speak to each other? b) Is it safe for children to play in the street during the day? c) Can you trust the people of the area? d) Are there good places to spend your free time? e) Are there many places for entertainment in the evening? f) Is there often violence and theft? g) Is it a beautiful area? h) Is it an isolated area? i) Do you have good public services (health center, youth center, etc.)?	a) Yes; b) No

To a better understanding of the results there was a need to recode and add variables, as observed on table 2. Variables were issued from HBSC questionnaires items described earlier. Items related to sports (Football, Basketball, Gymnastics, Volleyball, Swimming, Cycling/Mountain biking, Handball, Athletics) were added together. The Alpha Cronbach method was previously used to verify the internal consistence among these variables and obtained a weak (0.6) but acceptable value regarding internal consistency [32].

**Table 2:** Construction of variables and their coding

Questions	cod.
Sports practice (alpha: 0.6) a) Football, b) Basketball, c) Gymnastics, d) Volleyball, e) Swimming, f) Cycling/mountain biking, g) Handball, h) Athletics	0-1 (0 - no practice, 1-practicing)

Sports (KMO: 0.8): - "Indoor": b) Basketball, c) Gymnastics, d) Volleyball, g) Handball. - "Outdoor": a) Football, f) Cycling/mountain biking, h) Athletics	0-1 (0 - no practice, 1-practicing)

Characteristics of the neighborhood (KMO: 0.7) - Social characteristics: a) Do people get along well and speak to each other?; b) Is it safe for children to play in the street during the day?, c) Can you trust the people of the area?; f) Is there often violence and theft? - Physical Characteristics: d) Are there good places to spend your free time?; e) Are there many places for entertainment in the evening? g) Is it a beautiful area? i) Do you have good public services (health center, youth center, etc.)?	0-1 (0- no; 1- yes)

Regarding the items related to types of sports practice, two factors were identified by means of a factorial analysis using the method of the main components with Varimax rotation (referring to indoor or outdoor practice) which explained 40% of the variance, (eigen value 2.2). The two factors were therefore considered.

Regarding items related to neighborhood conditions, two items related to environmental characteristics of the neighborhood were found (social and physical characteristics) which explained 37.8% of the variance (eigen value 2). The two factors were therefore considered.

### Data analysis

The standard deviation and frequency (%) were calculated for the statistical analysis. Chi-squared test (χ2) was performed for comparison of frequencies between genders and school grade at the 0.05 level of significance. The first analysis focused on the interaction of gender and school grade on physical activity, sports, indoor sports and outdoor sports.

The second analysis focused on the explanatory models of practice of different types of physical practice through linear regression analysis with a 95% confidence interval.

Multivariate logistic regression analysis was performed to evaluate the strength of the association to each type of practice, adolescents (gender, school grade) and neighborhood characteristics. The calculations of the odds ratio (OR) and its 95% confidence interval (CI) were performed by SPSS (version 15.0).

## Results

The table 3 presents the distribution of the variables by frequency in analysis. 42% of the Portuguese students refer that they practice 3 to 5 times a week physical activity. In the matter of exercise, the most indicated category is 1 to 3 times a week (47,2%). There are 17,1% individuals who exercise once or less per month. Concerning sports practice, 42,6% reveal to exercise in indoor sports infrastructures and 50,1% outdoor.

**Table 3:** Descriptive statistics

Variable	n	%
Physical activity		
Not practicing	219	4,6
Less than 3 days/week.	1638	34,2
3 to 5 days/week.	2010	42
6 or more days/week	920	19,2

Sports practice		
1 time or less/month	816	17,1
1 to 3 days/week.	2245	47,2
More than 3 days/week.	1700	35,7
Practice of indoor sports	2080	42,6
Practice of outdoorsports	2444	50,1

Presence of features of the neighborhood		
Do people get along well and speak to each other?	4229	86,7
Is it safe for children to play in the street during the day?	3706	78,6
Can you trust the people of the area?	3643	77,6
Are there good places to spend your free time?	3565	75,8
Are there many places for entertainment in the evening?	1871	39,8
Is there often violence and theft?	928	19
Is it a beautiful area?	3653	78,2
Is it an isolated area too?	981	21
Do you have good public services (health center, youth center, etc.)?	2759	59,2

Teenagers were asked about the particularities of the place where they live and the majority referred the existence of positive characteristics. On the other hand, negative aspects of the place were also mentioned, like the existence of much violence and robbery (19%) and a too isolated area (21%).

The type of physical activity practiced by the teenagers was studied bearing in mind the gender and school grade distinction (see table 4). Regarding the practice of physical activity and exercise, females and older adolescents (10th grade) reported higher levels of practice. The specification of the practice in an indoor context seems to please more the female gender and students of the 6th grade. The practice of outdoor sports is more evident in the male gender and the 8th grade.

**Table 4:** Inference of the type of physical practice in accordance with the school grade and gender

	Variables	n	Gender (%)		School grade (%)		
			
			Boys	Girls	6th	8th	10th
Physical activityy	Not practicing	219	36,5*	63,5*	18,7*	33,8	47,5*
	less than 3 days/week.	1638	33*	67*	28,1*	36,3	35,6*
	3 to 5 days/week.	2010	54*	46*	29,4*	36,8	33,8
	6 or more days/week	920	71,6*	28,4*	44,3*	32,3*	23,4*

Sports	1 time or less/month	816	23,5*	76,5*	20*	33,1	46,9*
	1 to 3 days/week.	2245	44,9*	55,1*	29*	36,9	34,1
	More than 3 days/week.	1700	67,8*	32,2*	40,1*	35,5	24,4*

	Indoor sports	2080	45,8*	54,2*	38,1*	37,6*	24,3*
	Outdoor sports	2444	67,7*	32,3*	37,8*	38*	24,2*

*χ^2^; p < .005;

The table 5 shows the results of the multiple regression statistics where we can identify variables which are a significant predictor for the different types of physical practices of the adolescents.

**Table 5:** Explanatory models of practice of different types of physical practice

Variable to explain	variables included	β	*t*	*p*	**R**^**2**^_**a**_
**Physical activity**	Constant	5.378	36.747	.000	**0.108**
	**Gender**	-1.083	-18.636	.000	
	**School grade**	-0.333	-9.114	.000	
	**Are there are good places to spend your free time?**	0,205	2,956	.003	
	Do you have good public services?	0,176	2.95	.003	
	Is it safe for children to play in the street during the day?	0,202	2.832	.005	

**Sports practice**	Constant	1.41	11.699	.000	**0.137**
	**Gender**	.999	20.562	.000	
	**School grade**	.313	10.24	.000	
	**Are there are good places to spend your free time?**	-.213	-3.616	.000	
	Is it safe for children to play in the street during the day?	-0.2	-3.372	.001	
	Are there many places for entertainment in the evening?	-0.2	-3.892	.000	

**Indoor sports**	Constant	..752	10.894	.000	**0.03**
	**Gender**	0.097	3.27	.001	
	**School grade**	-0.176	-9.479	.000	
	**Are theree good places to spend your free time?**	0.103	2.877	.004	
	Are there many places for entertainment in the evening?	0.094	2.99	.003	

**Outdoor Sports**	Constant	1.5.78	29.234	.000	**0.155**
	**Gender**	-0.499	-22.89	.000	
	**School grade**	-0.155	-11.311	.000	
	**Are there good places to spend your free time?**	0.085	3.277	.001	
	Is it safe for children to play in the street during the day?	0.076	2.881	.004	
	Are there many places for entertainment in the evening?	0.074	3.193	.001	

R^2^_a_- linear regression analyses

All the models explained a low proportion of the variance in the practice, especially in indoor sports (R^2^_a _= 0.03). The variables explained 15.5% of the variance in outdoor sports, and 13.7% of the variance in exercise.

Gender variables, school grade and the existence of good places to spend their free time as significant predicators of all the types of practices are very interesting. The variable "is it safe for children to play on the street" (except indoor sports) and "are there many night leisure places" (except for PA) are important indicators in all types of practices.

The table 6 shows the *odds ratio *adjusted from the regressive logistic statistic for the variables of gender, school grade and the characteristics of the place and the influences on the practices of different types of physical activity.

**Table 6:** Logistic regression for gender, years of education and characteristics of the neighborhood characteristics according to each type of practice

Variable	PA OR (95%CI)	Exercise OR (95%CI)	Indoor sports OR (95%CI)	Outdoor Sports OR (95%CI)
Gender				
Girl	1	1	1	1
Boy	3.0 [2.6-3,4]*	0.3 [0,3-0.4]*	0.7 [0.6-0.8]*	4.7 [4.1-5.3]*

School grade				
10th	1	1	1	1
6th	1.7 [1.4-2.0]*	0,4 [0.4-0.5]*	2.2 [1.8-2.5]*	2.5 [2.1-2.9]*
8th	1.3 [1.1-1.5]*	0.6 [0.5-0.7]*	1.7 [1.5-1.9]*	2.1 [1.8-2.4]*

Do people get along well and speak to each other?				
Yes	1	1	1	1
No	0.9 [0.7-1,1]	1.1 [0.8-1.4]	0.9 [0.8-1.2]	0.9 [0.7-1.2]

Is it safe for children to play in the street during the day?				
Yes	1	1	1	1
No	0.8 [0.7-0.9]*	1.3 [1.1-1.5]*	0.9 [0.8-1.1]	0.8 [0.7-0.9]*

Can you trust the people of the area?				
Yes	1	1	1	1
No	1.2 [1.1-1.5]*	1.0 [0.8-1.2]	1.0 [0.8-1.1]	0.9 [0.7-1.0]

Are there good places to spend your free time?				
Yes	1	1	1	1
No	0.8 [0.7-0.9]*	1.3 [1.1-1.5 ]*	0.9 [0.7-1.0]	0.8 [0.7-0.9]*

Are there are many places for entertainment in the evening?				
Yes	1	1	1	1
No	0.9 [0.8-1.0]	1.2 [1.1-1.4]*	0.9 [0.8-1.0]	0.8 [0.7-0.9]*

Is there often violence and theft?				
Yes	1	1	1	1
No	0.9 [0.8-1.1]	0.8 [0.7-0.9]*	1.0 [0.8-1.2]	1.2 [1.0-1.5 ]*

Is it a beautiful area?				
Yes	1	1	1	1
No	1.0 [0.8-1.3]	0.9 [0,7-1.1]	1.0 [0.8-1.2]	0.8 [0.7-0.9]*

Is it an isolated area?				
Yes	1	1	1	1
No	0.9 [0.8-1.3]	1.0 [0.8-1.1]	1.0 [0.9-1.2]	1.0 [0.9-1.2]

Do you have good public services (health center, youth center, etc.)?				
Yes	1	1	1	1
No	0.8 [0.7-0.9]*	1.1 [1-1.3]	0.8 [0.7-0.9]*	1.1 [1.0-1.3]

	0,121	0,121	0,05	0,228
; *p*	5,92;0,65	0,12;0,76	5,12;0,74	23,68;0,03

Note: OR means *odds ratio; R*^*2*^_*N *_- *Nagelkerke *test; χ^2^_HL _- *Hosmer *e Lemeshow test; *p <*0.05*

The probability of adolescents doing physical activity seems to be linked to the male gender (*OR *= 3; *p *< .005), the 6th school grade (*OR *= 1.7; *p *< .005) or the 8th (*OR *= 1.3; *p *< .005) and the feeling that we cannot trust people (*OR *= 1.2; *p *< .005). The place is not safe (*OR *= 0.8; *p *< .005), there is no place to spend the free time (*OR *= 0.8; *p *< .005) as well as the neighborhood not having good public services (*OR *= 0.8; *p *< .005) condition the practice of physical activity.

The analysis of the practice of exercises show different variables than those referred in the practice of physical activity. Males (*OR *= 0.3; *p *< .005) and younger adolescents (6th grade - *OR *= 0.4; *p *< .005; 8th grade - *OR *= 0.6; *p *< .005) had a lower probability of exercising.

The perception that the neighborhood is unsafe induces the practice of exercise (*OR *= 1.3; *p *< .005), not having a place to spend the free time (*OR *= 1.3; *p *< .005) and not having many night leisure places (*OR *= 1.2; *p *< .005) contribute to the probability of the practice of exercise. The perception that in the neighborhood there is much violence and robbery conditions the practice (*OR *= 0.8; *p *< .005).

The indoor sports are practiced less in the male gender (*OR *= 0.7; *p *< .005) but attending 6th grade (*OR *= 2.2; *p *< .005) is linked to the practice of these sports. Regarding the neighborhood characteristics the only significant variable found is the non existence of good public services (*OR *= 0.8; *p *< .005) which limit the possibility of practice.

The option for outdoor sports seems to favor the male gender (*OR *= 4.7; *p *< .005) and the younger groups (6th grade - *OR *= 2.5; *p *< .005; 8th grade - *OR *= 2.1; *p *< .005). The practice of this type of sports seems to be more sensible to the neighborhood characteristics. The variables related to security, like the place not being safe (*OR *= 0.8; *p *< .005), violence and robbery (*OR *= 1.2; *p *< .005) condition the practice. The design of the neighborhood is very important. It is related to the less practice particularly when teenagers evaluate the place as being ugly (*OR *= 0.8; *p *< .005). The lack of night life leisure places (*OR *= 0.8; *p *< .005) and the lack of places to spend the free time (*OR *= 0.8; *p *< .005) conditions the possibility of practicing sports outdoor.

## Discussion

The present study intends to understand the importance of residential neighborhood in the practice of different physical activities and sports of Portuguese teenagers. The valorization of the ecological variables has resulted in a deeper investigation in the area, showing some guided tendencies which are not yet consensual. Besides that, most investigations have tried to explain the habitual physical activity, forgetting sports and specific types of sports. This study seeks to understand the phenomena linked to the practice of physical activity and also the practice of exercise and indoor as well as outdoor sports.

In the many statistical procedures undergone, the demographic questions (gender and age) are consistent in their influence of diverse types of practices which are commonly referred in the literature [30,33,34]. However, significant differences can be seen in the preferences of the genders related to the practice. The male gender and the younger ones state that they practice all the options in the study except the indoor activities. In the technical regressive logistic statistic we found a reduction of the practice of exercise regarding the male gender. This fact may be because of the type of sports that may be done in indoor infrastructures, once they can be more individual and with less physical contact among players [35], not exposed to natural weather conditions [34] and other aspects related to security [22].

In all the explained models of the practice obtained by the linear regression technique, the demographic questions and the existence of good places to spend the free time revealed a bigger importance. This evidence confirms the opinion of authors [9,20,25] that consider the accessibility of the leisure places an important stimulus for the practice. This aspect assumes a greater importance among the teenagers aged from 12 to 15 years old, for they already have some autonomy to go on foot or on their bicycle alone or with their friends [13]. For Kahn et al. [33] the lack of transport to the leisure place is linked to the physical inactivity.

The results of the regressive logistics of the various types of practices are quite interesting, particularly the questions related to security. Perception that the place is not safe for the children to play seems to be related to the practice of physical activity and the outdoor sports and raises the practice of exercise. This tends to transmit that the type of practice may be influenced by this perception, so as to lead teenagers to seek more guided and safe activities [20].

Trust in the people is another aspect that is linked to the perception of security. When we feel that in the neighborhood there are people who we can trust, it raises the practice of physical activity (to the other sports activities there was no link). This result contradicts the investigation of Romero [25] who considers that the presence of an adult transmits confidence and it is linked to the increase of practice of the teenagers. For the children's parents the social aspects of the neighborhood (the presence of trust worthy neighbors or their children having friends in the neighborhood that play together) are an important contribution to increase the indicators of confidence and consequently the increase of practice [36].

The place being beautiful seems to be important for the teenagers who practice activities outdoor. This valorization of the design aspect seems coherent, because these teenagers do activities with a strong and sensible link to the environment, like in the practice of athletics. For Mota et al. [10] the design aspects of the neighborhood are linked to more practices of physical activity. The study of Evenson et al. [22], with results from 610 girls from the USA, uncovered that the design aspects, i.e., the existence of trees, things to see, beautiful scenarios, the inexistence of trash are not associated to the rise of active transport to school, like to walk or to ride the bicycle, but it is positively linked to the practice of PA.

The present study has a number of limitations; first, there was a reliance on self-reported measures which do not provide accurate prevalence estimates; second, the fact that the questions used are not only directed to the practice of the physical activity, which could have led to some difficulties of interpretation of those who answered the questionnaire; third the cross-sectional study design conditioned the typology of the questions, fourth the reliability and validity of neighborhood measures.

The conclusion of this study leads to the presumption that the social and physical characteristics of the place are important for the practice of physical activity and sports. The security issue of the neighborhood and the existence of quality places are effective factor for the practice, so safety measures should be studied and the reinforced. The specificity of the type of practice and its associated environmental variables should continue to be studied in order to manage the definition of specific guidelines for each context.

## Competing interests

The authors declare that they have no competing interests.

## Authors' contributions

NL has substantially contributed to conception and design of the study, to analysis and interpretation of data, involved in drafting the manuscript, acted as the corresponding author, has read and approved the final manuscript.

MGM has substantially contributed to conception and design of the study, to analysis and interpretation of data, involved in drafting the manuscript, has read and approved the final manuscript.

MMS has substantially contributed to conception and design of the study, to analysis and interpretation of data, involved in drafting the manuscript, has read and approved the final manuscript.

JM has substantially contributed to conception and design of the study, to analysis and interpretation of data, involved in drafting the manuscript, has read and approved the final manuscript.

JAD has substantially contributed to conception and design of the study, to analysis and interpretation of data, involved in drafting the manuscript, has read and approved the final manuscript.
